# Surprisal and Valuation in the Predictive Brain

**DOI:** 10.3389/fpsyg.2012.00415

**Published:** 2012-10-17

**Authors:** Bryce Huebner

**Affiliations:** ^1^Department of Philosophy, Georgetown UniversityWashington, DC, USA

Clark (in press) argues that perception and action depend on “hierarchical predictive coding” systems, which attempt to reduce surprisal (a measure of the implausibility of a state given a model of the world). But, his appeal to surprisal-reduction does not explain the motivation to seek change, initiate motion, or engage in exploration. As he notes, “staying still inside a darkened room would afford easy and nigh-perfect prediction of our own neural states” (Clark, in press, p. 37). Clark claims that inborn expectations yield instinctual and tropistic behavior; and, he is right that surprisal-reduction mechanisms could modify behavior and reduce discrepancies between outcomes and these expectations. But, biological organisms must also recognize that strategies can be *better* and *worse*; and, they must be able to update their goals when the *value* of a reward changes (e.g., as they become sated or hungry). Even on the assumption that cortical processing aims to minimize prediction-errors, processes like learning, motivation, and decision-making also require valuation.

The location and stability of food and water are often uncertain. So, intelligent foraging requires evaluative strategies that can determine which practices are likely to yield the best payoffs relative to the costs of acting (Montague et al., 2012). Savvy organisms should act when the benefits are likely to outweigh the costs of seeking change and engaging in exploration (Montague and King-Casas, 2007). But, this situation is complicated by the fact that dangerous and unforeseen situations often require making rapid decisions that are sensitive to the *cost of acting* as well as the *value of the payoff* that can be expected in pursuing a reward. This is why savvy organisms must possess mechanisms that facilitate reward-seeking where payoffs are better than previously experienced. But, this requires treating outcomes and strategies as *better* and *worse*, which requires more than just minimizing prediction-errors.

Although there is debate over the precise mechanisms responsible for *valuation*, a broad consensus has emerged that one core mechanism is implemented by a network of midbrain dopaminergic neurons that compute prediction-error signals for expected rewards. This network computes a bi-directional teaching signal, which monitors the extent to which outcomes are better or worse-than-expected. Spiking rates in the basal ganglia, for example, increase when rewards are better-than-expected, decrease when they are worse-than-expected, and are unaffected when the time and quantity of rewards is accurately predicted (Montague et al., 1996). These evaluative error signals are computed for primary rewards; and, they can be attuned to respond to almost any reward-predicting stimuli – suggesting that they compute a polysensory and multimodal signal that can direct attention, learning, and action-selection in light of various valuable outcomes (Schultz, 1998, 2010). Curiously, these mechanisms also respond to novel events independently of their value; but, there is reason to suppose that this is because dopaminergic signals motivate exploration by treating novelty as its own reward (Liljeholm and O’Doherty, 2012).

Similar evaluative mechanisms seem to be found throughout the brain. For example, mechanisms in the ventral striatum compute expectations when the distribution and likelihood of a reward is uncertain; and there are distinct circuits in the ventral striatum and anterior insula that evaluate risk and compute risk-prediction-error signals (Preuschoff et al., 2006, 2008; Quartz, 2009). Similar mechanisms in ventral caudate seem to implement “fictive error” signals, which compare actual outcomes against “things that could have been,” thus allowing organisms to update their expectations in light of imagined feedback (Lohrenz et al., 2007). Finally, evaluative mechanisms in orbitofrontal cortex represent reward values – in concert with mechanisms in the basal ganglia – in a way that seems to facilitate making choices on the basis of the probability of a positive outcome, given recent patterns of gains and losses (Frank and Claus, 2006). Together, these types of evaluative mechanisms appear to implement the learning signals and motivational “umph” required to get Pavlovian, habitual, and goal-directed learning off the ground (Rangel et al., 2008; Liljeholm and O’Doherty, 2012).

I contend that we also need evaluative processes to understand how cultural practices “stack the dice so that we can more easily minimize costly prediction-errors” (Clark, in press, p. 43). Evaluative mechanisms can facilitate *cultural attunement* by treating norm compliance as rewarding and norm violation as aversive (Montague, 2006). And, perceived deviations from social norms appear to evoke neural responses that are similar to prediction-error signals (Klucharev et al., 2009, 2011). But, why would these prediction-error signals ever lead us to revise social institutions and social practices, as opposed to leading us to recalibrate our judgments? A purely calibrational mechanism can make sense of the conservative aspects of habitual learning and cultural attunement, but they leave the (relatively rare) cases where people attempt to reconfigure their environments in ways that better suit their interests mysterious.

We need an account of valuational mechanisms to understand these practices of social niche construction. The decision to change your environment is always risky. And, risky decisions require not only the ability to predict rewards, but also to evaluate the likelihood of success and the value of achieving your goals. It may be possible to get genuine norm compliance from a system that doesn’t represent value – though I am skeptical. But, deciding to reject a norm, to challenge a social institution, or to develop better practices requires *evaluating* the likely outcomes as better and worse. Surprisal-reduction mechanisms cannot represent things as better and worse, they can only represent and reduce deviations from our expectations. However, constructing a world that stacks the dice in our favor sometimes requires pursuing a world that is better than the one we expect.
